# Sphingolipids in Congenital Diaphragmatic Hernia; Results from an International Multicenter Study

**DOI:** 10.1371/journal.pone.0155136

**Published:** 2016-05-09

**Authors:** Kitty G. Snoek, Irwin K. M. Reiss, Jeroen Tibboel, Joost van Rosmalen, Irma Capolupo, Arno van Heijst, Thomas Schaible, Martin Post, Dick Tibboel

**Affiliations:** 1 Intensive Care and Department of Paediatric Surgery, Erasmus Medical Center- Sophia Children’s Hospital, Rotterdam, The Netherlands; 2 Department of Neonatology, Erasmus Medical Center- Sophia Children’s Hospital, Rotterdam, The Netherlands; 3 Department of Biostatistics, Erasmus Medical Center, Rotterdam, The Netherlands; 4 Department of Medical and Surgical Neonatology, Bambino Gesù Children’s Hospital, Rome, Italy; 5 Department of Neonatology, Radboud University Medical Centre, Nijmegen, The Netherlands; 6 Department of Neonatology, Universitätsklinikum Mannheim, Mannheim, Germany; 7 Program of Physiology and Experimental Medicine, Hospital for Sick Children Research Institute, Toronto, Canada; University of Giessen Lung Center, GERMANY

## Abstract

**Background:**

Congenital diaphragmatic hernia is a severe congenital anomaly with significant mortality and morbidity, for instance chronic lung disease. Sphingolipids have shown to be involved in lung injury, but their role in the pathophysiology of chronic lung disease has not been explored. We hypothesized that sphingolipid profiles in tracheal aspirates could play a role in predicting the mortality/ development of chronic lung disease in congenital diaphragmatic hernia patients. Furthermore, we hypothesized that sphingolipid profiles differ between ventilation modes; conventional mechanical ventilation versus high-frequency oscillation.

**Methods:**

Sphingolipid levels in tracheal aspirates were determined at days 1, 3, 7 and 14 in 72 neonates with congenital diaphragmatic hernia, born after > 34 weeks gestation at four high-volume congenital diaphragmatic hernia centers. Data were collected within a multicenter trial of initial ventilation strategy (NTR 1310).

**Results:**

36 patients (50.0%) died or developed chronic lung disease, 34 patients (47.2%) by stratification were initially ventilated by conventional mechanical ventilation and 38 patients (52.8%) by high-frequency oscillation. Multivariable logistic regression analysis with correction for side of the defect, liver position and observed-to-expected lung-to-head ratio, showed that none of the changes in sphingolipid levels were significantly associated with mortality /development of chronic lung disease. At day 14, long-chain ceramides 18:1 and 24:0 were significantly elevated in patients initially ventilated by conventional mechanical ventilation compared to high-frequency oscillation.

**Conclusions:**

We could not detect significant differences in temporal sphingolipid levels in congenital diaphragmatic hernia infants with mortality/development of chronic lung disease versus survivors without development of CLD. Elevated levels of ceramides 18:1 and 24:0 in the conventional mechanical ventilation group when compared to high-frequency oscillation could probably be explained by high peak inspiratory pressures and remodeling of the alveolar membrane.

## Introduction

In patients with congenital diaphragmatic hernia (CDH), lung related problems such as chronic lung disease (CLD) and pulmonary vascular disease including pulmonary hypertension are the primary causes of mortality [1] with ventilator-induced lung injury (VILI) and high concentrations of oxygen predisposing newborns to develop CLD as prime morbidity [2].

Since 2008, CDH neonates are treated according to the same neonatal treatment protocol, developed during a consensus meeting of the CDH EURO Consortium [3]. Prenatally diagnosed CDH infants are intubated after birth and mechanically ventilated. Conventional mechanical ventilation (CMV) and high-frequency oscillation (HFO) are two ventilation modalities that are associated with VILI and thus predispose to developing CLD. In a randomized clinical trial (the VICI-trial) looking at initial ventilation strategy (CMV vs HFO) of prenatally diagnosed CDH infants, 41 of the 91 patients (45.1%) initially ventilated with CMV died or had CLD by day 28 compared to 43 of the 80 patients (53.8%) using HFO [4]. This difference, however, did not reach statistical significance.

The exact mechanism for the development of CLD in CDH remains unknown. In postmortem lung biopsies non-specific features as persistent inflammation, edema, vascular changes and parenchymal fibrosis were observed after mechanical ventilation [5]. Due to changes in treatment modalities over the years in premature born neonates, the so-called ‘new BPD’ developed, characterized by interrupted septation and abnormal vascularization, leading to fewer and enlarged alveoli [6]. In contrast to premature born neonates, lungs of fetuses with CDH are not surfactant deficient [7], and surfactant replacement therapy has no beneficial effect in term neonates with CDH [8].

Sphingolipids are classically thought to be purely structural elements of the cell membrane, but have been revealed as key bioactive mediators in a variety of pathophysiological processes [9]. They have an important role as messenger molecule in the regulation of proliferation and apoptosis [10]. Sphingolipids are involved in lung development, injury and repair as suggested by elevated sphingolipid levels in brochoalveolar lavage of newborn rats exposed to hyperoxia (injury model of CLD) which declined during subsequent ambient air exposure (repair) [11]. Various sphingolipids play an important role in cellular homeostasis. Ceramide leads to cell-cycle arrest and apoptosis, whereas sphingosine-1-phosphate is involved in facilitating proliferation and differentiation of cells. The interplay between pro-apoptotic and apoptotic ‘rheostat’ determined the formation of lung structure, both macroscopically and on a cellular level, during all stages of lung development [12].

Given the lack of knowledge on the pathogenesis of CLD in CDH, we have analyzed the bronchoalveolar lavage for sphingolipids in tracheal aspirates at specific time-points during the first month of a prospective ventilation study (VICI-trial) [4]. We hypothesized that sphingolipid profiles could have a predictive role for mortality/ development of CLD. Secondly, we aimed to determine whether CMV versus HFO ventilation leads to different sphingolipid levels in CDH patients. These aims were achieved.

## Materials and Methods

### Patient Population

Inborn neonates born between November 2008 and December 2013, after a gestation of more than 34 weeks with a prenatal diagnosis of CDH, were included in a multicenter RCT of initial ventilation strategy (NTR 1310)(4). Ethical approval was given by the medical ethics review board of Erasmus MC, Rotterdam, the Netherlands (NTR 1310). Thereafter, all local medical ethical committees gave their approval (Bambino Gesu Children's Hospital, Universitätsklinikum Mannheim, Radboud University Medical Centre). Parents gave written informed consent. The procedures, including obtaining informed consent, were conducted in accord with the ethical standards of the Committee on Human Experimentation of the institution in which the experiments were done. Exclusion criteria were: severe chromosomal anomaly such as trisomy 13 or 18, which may imply a decision to stop or not to start medical treatment; severe cardiac anomalies expected to need corrective surgery in the first 60 days after birth; renal anomalies associated with oligohydramnios; severe orthopedic and skeletal deformities which were likely to influence thoracic or lung development; and severe anomalies of the central nervous system. Patients with a gestational age of less than 34 weeks were excluded so that the results could not be influenced by severe lung prematurity. Written parental informed consent was obtained before birth. All children were treated according to the same standardized protocol [3]. Patients were randomized for initial ventilation strategy (CMV or HFO) within two hours after birth. CLD was defined as oxygen dependency (>21%) at day 28 as described by Jobe and Bancalari [13]. Diaphragmatic defect size was classified according to the definition of the CDH study group and assessed during surgical repair [14].

### Tracheal Aspirates

Tracheal aspirates were obtained during routine tracheal suctioning within the first 24 hours of life and at day 3, 7, and 14. Tracheal aspirates were only collected during the period of mechanical ventilation. During tracheal suctioning, flushing with 0.5–1.0 ml saline was performed according to standard practice. Tracheal aspirates were immediately centrifuged at 1500g for 6 minutes at 20°C and samples were stored at -80°C until analysis.

### Sphingolipid measurements

Sphingolipids were measured as previously described [11]. Briefly, lipids were extracted from tracheal aspirate samples and sphingolipids were separated and analyzed using high performance liquid chromatography and tandem mass spectrometry (LC-MS/MS). The analyses were performed at the Analytical Facility for Bioactive Molecules of the Hospital for Sick Children, Toronto, ON, Canada. Sphingolipid levels were presented as ng/mL.

### Statistical analysis

Patient characteristics were described as number (%) for categorical variables, mean ± SD for normally distributed variables or median (interquartile range; IQR) for continuous variables that were not normally distributed. Patient characteristics between participants and non-participants were analyzed using independent samples t-test for continuous data or chi square tests for categorized data. Sphingolipid levels were compared between patients who died/ developed CLD and patients who survived/ did not develop CLD, and between patients who were initially ventilated by CMV and HFO by using the Mann-Whitney U test. All analyses were performed for each of the four time points separately. Independent associations between sphingolipids levels and mortality/ development of CLD were determined using multivariable logistic regression modelling and were presented as odds ratio (OR) [95% confidence interval], *p*-value. Observed-to-expected lung-to-head ratio (O/E LHR), side of the defect, liver position, center and ventilation mode were included as independent variables in these models. Sphingolipid levels were logarithmically transformed due to skewed distribution, and non-detectable values were set to the lower detection threshold 0.03 ng/mL. The calibration of the multivariable logistic regression models was assessed using the Hosmer-Lemeshow goodness-of-fit test. All statistical tests, except for the analyses to determine difference in patients characteristics between participants and non-participants, were two-sided and used a Bonferroni-adjusted significance level of 0.0125 to correct for multiple testing at the four time points. All analyses were performed using SPSS version 21.0 for Windows statistical software.

## Results

Tracheal aspirates were collected in 69 patients of the 171 infants who were included in the RCT at the four participating centers. Additionally, in three patients from one center there was only written consent for sample collection, and not for randomization of initial ventilation mode. Thus, tracheal aspirates from 72 patients were collected at various time-points. 179 tracheal aspirates were obtained, of which 49 were collected at day 1, 56 samples at day 3, 46 samples at day 7, and 21 samples at day 14. Patients who had tracheal aspirates collected showed an increased prevalence of left-sided diaphragm defect compared to the patients who had no aspirates collected (Table 1). Of the 72 included patients, 16 patients (22.2%) died in the first year of life, and 20 of the 56 survivors (35.7%) developed CLD; thus, 36 patients (50.0%) died/ developed CLD. Thirty-eight patients (52.8%) were initially ventilated by HFO and 34 patients (47.2%) by CMV.

**Table 1:** Patient characteristics.

	Included patients n = 72	Non-included patients n = 102	p-value
**Gestational age (weeks)**	38.0± 1.2	37.9± 1.4	0.66
**Birth weight (grams)**	2957± 443	2901± 467	0.43
**Fetoscopic endotracheal occlusion**	5 (6.9%)	14 (13.7%)	0.16
**Male gender**	33 (45.8%)	51 (50.0%)	0.59
**Left sided defect**	68 (94.4%)	83 (81.4%)	0.01
**Liver position: intrathoracic**	37 (51.4%)	66 (64.7%)	0.08
**Type of repair**			0.06
Patch correction	48 (66.7%)	46 (45.1%)	
Primary closure	19 (26.4%)	35 (34.3%)	
No repair	5 (6.9%)	21 (20.6%)	
**Diaphragmatic defect size**			0.05
A	5 (6.9%)	7 (6.9%)	
B	5 (6.9%)	28 (27.4%)	
C	21 (29.2%)	34 (33.3%)	
D	35 (48.7%)	10 (9.8%)	
No repair	5 (6.9%)	21 (20.6%)	
Treatment with nitric oxide	33 (45.8%)	53 (52.0%)	0.43
ECMO (if ECMO was available)	22/ 56 (39.3%)	20/ 55 (36.4%)	0.76
Treatment with inotropics	60 (83.3%)	89 (87.2%)	0.47
Age at repair (days)	5.0 (3.0–9.0)	4.0 (3.0–6.0)	0.28
Ventilation time (days)	15.0 (7.8–23.0)	10.0 (7.0–17.5)	0.29
ICU admission (days)	22.5 (15.3–39.8)	20.5 (13.0–40.5)	0.99

Data are presented as n (%), mean± SD or median (IQR). Abbreviations: ECMO: extracorporeal membrane oxygenation. HFO: high-frequency oscillation; ICU: intensive care unit.

**Fig 1:**
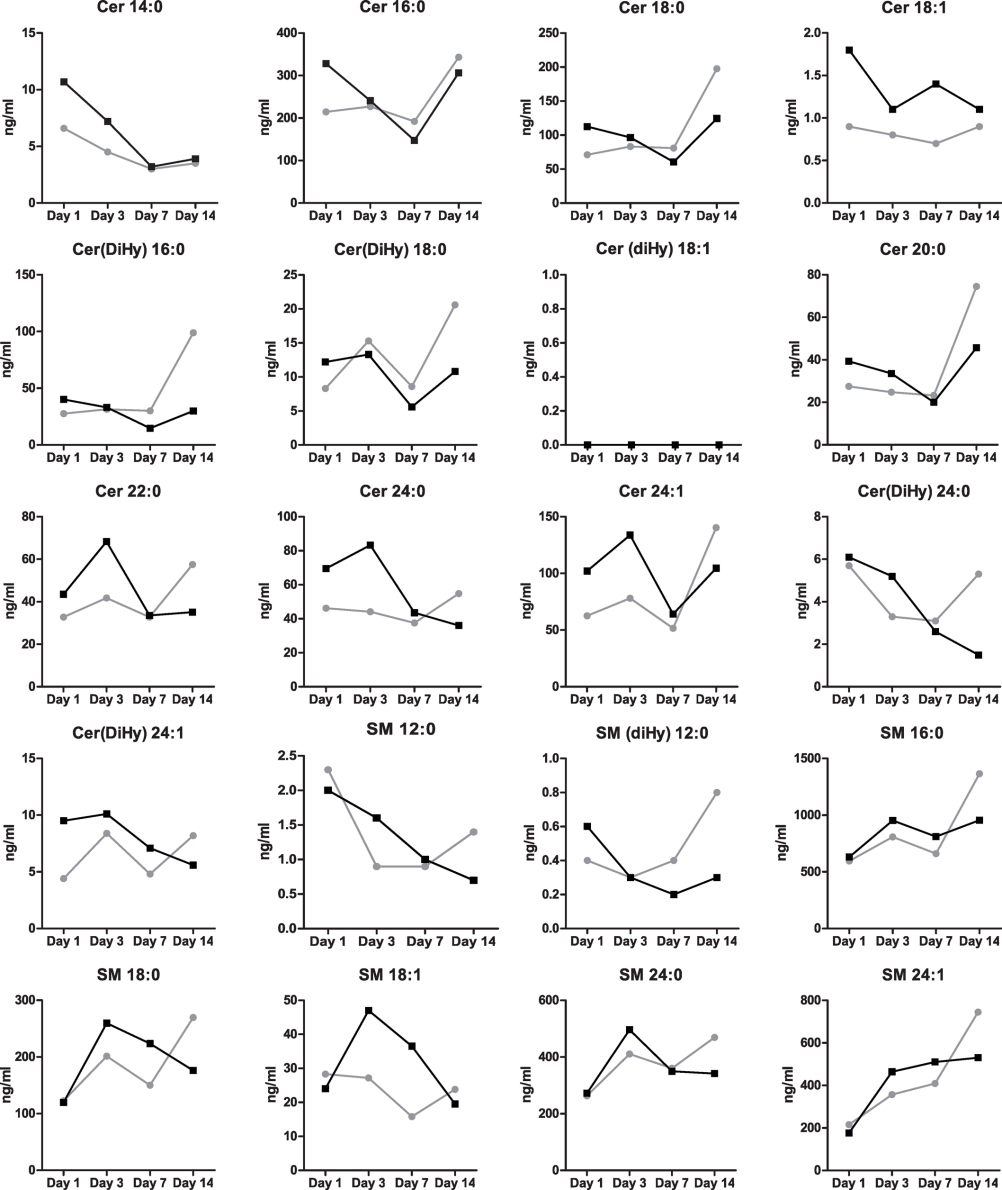
Median values of sphingolipids over time. Grey: No CLD or alive. Black: CLD or died.

No significant differences in sphingolipid profiles were found at day 1, 3, 7 and 14 between patients who died/ developed CLD and patients who survived/did not develop CLD (Table 2). Conclusions were not different after exclusion of the five FETO-treated patients (results not shown). Median sphingolipid levels over time for each sphingolipid are presented in Fig 1. Multivariable logistic regression analysis with correction for O/E LHR, side of the defect, liver position, center and ventilation mode showed that none of the sphingolipid levels were associated with mortality/development of CLD (Table 3). Conclusions were not different after exclusion of the five FETO-treated patients (results not shown). The p-values of the Hosmer-Lemeshow test were larger than 0.05, indicating an adequate model calibration.

**Table 2:** Associations of sphingolipids with outcome mortality/ development of CLD.

	*Day 1*		*Day 3*		*Day 7*		*Day 14*	
Independent variable	Median (IQR)	*p*-value	Median (IQR)	*p*-value	Median (IQR)	*p*-value	Median (IQR)	*p*-value
**Cer 14:0**		0.37		0.35		0.76		
No CLD or alive	6.6 (3.0–14.6)		4.5 (2.7–10.8)		3.0 (1.5–7.3)		3.5 (0.8–8.0)	
CLD or died	10.7 (5.1–20.0)		7.2 (2.6–14.5)		3.2 (0.9–13.2)		3.9 (1.9–8.7)	
**Cer 16:0**		0.70		0.73		0.43		0.97
No CLD or alive	214.5 (144.1–487.8)		227.0 (118.4–380.4)		192.0 (114.0–730.0)		343.0 (81.6–649.1)	
CLD or died	328.0 (120.0–612.0)		240.8 (133.9–504.8)		147.5 (48.0–565.0)		306.5 (122.5–781.9)	
**Cer 18:0**		0.86		0.90		0.50		0.90
No CLD or alive	71.2 (41.9–176.4)		83.2 (45.5–167.1)		81.0 (45.2–268.0)		197.5 (50.5–278.8)	
CLD or died	112.5 (33.7–265.5)		96.5 (43.5–164.8)		60.5 (15.5–183.5)		124.5 (37.0–339.4)	
**Cer 18:1**		0.57		0.78		0.61		0.64
No CLD or alive	0.9 (0.3–2.0)		0.8 (0.5–1.8)		0.7 (0.2–2.9)		0.9 (0.2–1.7)	
CLD or died	1.8 (0.1–3.5)		1.1 (0.4–2.7)		1.4 (0.1–3.4)		1.1 (0.4–2.5)	
**Cer(DiHy)16:0**		0.61		0.64		0.60		0.93
No CLD or alive	27.6 (14.1–73.0)		31.4 (15.4–60.0)		30.1 (12.4–53.0)		98.9 (1.3–196.5)	
CLD or died	40.0 (12.5–104.3)		33.1 (14.7–92.5)		14.7 (4.5–84.6)		29.9 (18.8–97.8)	
**Cer(DiHy)18:0**		0.67		0.94		0.53		0.52
No CLD or alive	8.3 (3.7–17.7)		15.3 (7.0–22.8)		8.6 (3.3–36.2)		20.6 (5.3–64.5)	
CLD or died	12.2 (2.8–31.3)		13.3 (7.3–31.5)		5.6 (2.1–26.3)		10.8 (5.7–29.5)	
**Cer(DiHy)18:1**		0.35		0.93		0.04		0.97
No CLD or alive	0.03 (0.03–0.03)		0.03 (0.03–0.03)		0.03 (0.03–0.03)		0.03 (0.03–0.56)	
CLD or died	0.03 (0.03–0.30)		0.03 (0.03–0.03)		0.03 (0.03–1.21)		0.03 (0.03–0.13)	
**Cer 20:0**		0.69		0.46		0.46		0.90
No CLD or alive	27.5 (15.6–67.9)		24.8 (14.1–52.2)		23.2 (15.0–94.5)		74.5 (16.3–105.5)	
CLD or died	39.3 (14.1–108.0)		33.6 (20.2–58.2)		20.0 (5.4–66.5)		45.7 (14.0–122.2)	
**Cer 22:0**		0.59		0.16		0.65		0.83
No CLD or alive	32.8 (18.0–88.5)		41.8 (14.6–67.9)		32.8 (16.0–137.5)		57.5 (14.7–109.9)	
CLD or died	43.5 (17.7–198.8)		68.3 (24.4–91.9)		33.6 (3.9–108.5)		35.1 (20.0–130.5)	
**Cer 24:0**		0.42		0.16		0.76		0.89
No CLD or alive	46.2 (24.7–122.5)		44.1 (17.1–105.0)		37.5 (14.4–217.5)		54.8 (14.6–114.6)	
CLD or died	69.5 (25.3–340.3)		83.3 (24.5–161.0)		43.5 (4.4–125.5)		36.0 (23.1–102.3)	
**Cer 24:1**		0.52		0.23		0.65		0.90
No CLD or alive	62.5 (36.1–174.5)		78.0 (27.5–157.3)		51.5 (29.9–449.5)		140.3 (32.3–343.4)	
CLD or died	102.0 (32.7–352.0)		133.8 (49.3–196.6)		64.0 (10.0–203.5)		104.5 (40.7–306.5)	
**Cer(DiHy)24:0**		0.96		0.31		0.48		0.41
No CLD or alive	5.7 (2.8–13.2)		3.3 (1.2–6.7)		3.1 (0.8–11.0)		5.3 (2.1–11.3)	
CLD or died	6.1 (2.5–22.8)		5.2 (1.6–12.2)		2.6 (0.3–11.4)		1.5 (1.0–8.9)	
**Cer(DiHy)24:1**		0.42		0.25		0.88		0.90
No CLD or alive	4.4 (1.6–16.2)		8.4 (2.5–15.4)		4.8 (1.2–29.7)		8.2 (1.3–39.9)	
CLD or died	9.5 (2.0–20.7)		10.1 (2.4–25.3)		7.1 (0.8–25.9)		5.6 (1.6–16.6)	
**SM12:0**		0.53		0.06		1.00		0.97
No CLD or alive	2.3 (1.2–3.7)		0.9 (0.5–1.8)		0.9 (0.3–2.2)		1.4 (0.2–3.2)	
CLD or died	2.0 (1.1–4.4)		1.6 (0.6–3.3)		1.0 (0.3–2.2)		0.7 (0.6–2.0)	
**SM(DiHy)12:0**		0.59		0.45		0.30		0.36
No CLD or alive	0.4 (0.3–0.7)		0.3 (0.2–0.5)		0.4 (0.2–0.6)		0.8 (0.2–1.5)	
CLD or died	0.6 (0.3–1.0)		0.3 (0.2–0.8)		0.2 (0.1–0.5)		0.3 (0.1–0.7)	
**SM16:0**		0.89		0.69		0.43		0.64
No CLD or alive	595.0 (355.1–1053.8)		808.5 (520.0–1247.5)		660.0 (497.5–1862.0)		1365.0 (311.9–1722.5)	
CLD or died	630.0 (300.5–1352.5)		953.3 (411.6.61–1701.3)		810.0 (122.5–1385.0)		955.0 (518.3–1577.5)	
**SM18:0**		0.75		0.62		0.82		0.97
No CLD or alive	122.5 (71.4–259.3)		201.3 (91.8–300.8)		150.0 (82.0–325.0)		269.5 (64.5–346.5)	
CLD or died	119.5 (60.0–296.5)		259.5 (86.3–387.5)		223.5 (30.9–360.0)		176.0 (103.8–409.9)	
**SM18:1**		0.93		0.30		0.65		0.97
No CLD or alive	28.3 (15.6–57.7)		27.2 (14.7–56.3)		15.8 (9.3–51.5)		23.8 (5.4–70.5)	
CLD or died	24.0 (12.4–85.5)		47.0 (14.3–119.1)		36.5 (4.0–82.1)		19.5 (8.0–41.7)	
**SM24:0**		0.91		0.59		0.73		0.97
No CLD or alive	263.0 (124.6–585.3)		410.5 (203.0–661.3)		360.0 (180.5–982.0)		469.0 (122.8–571.3)	
CLD or died	272.0 (122.8–761.0)		496.3 (241.0–797.0)		349.5 (69.5–1020.0)		341.5 (232.3–630.0)	
**SM24:1**		0.83		0.61		0.51		0.97
No CLD or alive	215.8 (102.4–410.7)		357.3 (187.1–680.0)		409.0 (183.5–1116.0)		745.0 (105.9–1278.8)	
CLD or died	176.5 (80.8–535.0)		464.0 (172.6–877.3)		510.0 (62.0–1025.0)		530.0 (219.0–1107.5)	

Sphingolipids were expressed as ng/mL. Abbreviations: CLD: chronic lung disease; IQR: interquartile range; Cer: ceramide; SM: sphingomyelin.

**Table 3:** Multivariable logistic regression analysis with outcome mortality/ development of CLD.

	DAY 1		DAY 3		DAY 7	
Independent variable	OR	95% CI	OR	95% CI	OR	95% CI
Cer 14:0	0.826	0.457–1.495	1.718	0.654–4.516	1.055	0.581–1.916
Cer 16:0	0.713	0.364–1.397	1.269	0.527–3.059	0.969	0.509–1.846
Cer 18:0	0.709	0.389–1.294	0.930	0.384–2.255	0.872	0.463–1.643
Cer 18:1	0.642	0.345–1.193	1.447	0.697–3.005	1.047	0.667–1.644
Cer(DiHy)16:0	0.452	0.135–1.511	2.854	0.519–15.694	0.970	0.470–2.001
Cer(DiHy)18:0	0.453	0.193–1.061	1.191	0.451–3.151	0.939	0.569–1.548
Cer(DiHy)18:1	1.433	0.710–2.893	1.040	0.469–2.304	1.267	0.704–2.282
Cer 20:0	0.692	0.358–1.337	1.016	0.428–2.407	0.893	0.481–1.660
Cer 22:0	0.744	0.400–1.385	1.290	0.605–2.750	0.964	0.568–1.634
Cer 24:0	0.802	0.456–1.410	1.531	0.681–3.442	0.957	0.635–1.442
Cer 24:1	0.801	0.459–1.399	1.369	0.645–2.902	0.978	0.587–1.628
Cer(DiHy)24:0	0.650	0.391–1.080	1.369	0.699–2.681	0.901	0.558–1.454
Cer(DiHy)24:1	0.787	0.497–1.245	1.175	0.653–2.113	1.059	0.753–1.489
SM12:0	0.611	0.270–1.380	1.446	0.444–4.705	1.116	0.641–1.943
SM(DiHy)12:0	0.942	0.362–2.448	0.936	0.323–2.717	0.880	0.401–1.932
SM16:0	0.444	0.142–1.382	0.750	0.212–2.654	0.917	0.489–1.720
SM18:0	0.496	0.186–1.324	0.924	0.363–2.349	0.934	0.473–1.846
SM18:1	0.754	0.418–1.361	1.019	0.461–2.252	1.123	0.639–1.973
SM24:0	0.542	0.214–1.374	0.958	0.369–2.483	0.920	0.529–1.601
SM24:1	0.636	0.311–1.300	0.988	0.394–2.477	0.919	0.520–1.624

Abbreviations: CLD: chronic lung disease; OR: odds ratio; CI: confidence interval; Cer; ceramide; SM: sphingomyelin. Observed-to-expected lung-to-head ratio, side of the defect, liver position, center and ventilation arm were included as independent variables. Day 14: too limited number of observations to perform multivariable logistic regression analysis.

No significant differences in sphingolipid profiles were found at days 1, 3, and 7 between patients who were initially ventilated by CMV versus HFO (Table 4). At day 14, ceramide-C18:1 and ceramide-C24:0 were increased for patients initially ventilated by CMV (median 1.4 (IQR 1.1–4.3))and (median 81.0 (IQR 33.6–205.2)) respectively compared to HFO (median 0.5 (0.0–1.1)) (p = 0.005) and (median 25.3 (IQR 2.9–53.0)) respectively (p = 0.008). In a selection of patients who died/ developed CLD, at day 14 ceramide-C18:1 was increased for patients initially ventilated by CMV (median 1.4 (IQR 1.2–5.6)) compared to HFO (median 0.5 (0.1–1.1)) (p = 0.009), but ceramide-C24:0 was significantly increased in patients initially ventilated by CMV (median 102.5 (IQR 31.3–225.9)) compared to HFO (median 23.3 (IQR 6.4–33.7)) (p = 0.011).

**Table 4:** Sphingolipid levels for initial ventilation (CMV or HFO).

	*Day 1*		*Day 3*		*Day 7*		*Day 14*	
Independent variable	Median (IQR)	*p*-value	Median (IQR)	*p*-value	Median (IQR)	*p*-value	Median (IQR)	*p*-value
**Cer 14:0**		0.98		0.54		0.90		0.05
CMV	7.8 (3.0–17.4)		6.2 (2.7–13.7)		0.3 (1.1–13.2)		6.9 (2.9–11.0)	
HFO	8.2 (3.6–18.0)		5.2 (2.3–13.7)		3.2 (0.9–9.4)		2.6 (0.4–4.6)	
**Cer 16:0**		0.98		0.44		0.68		0.06
CMV	266.5 (112.0–534.8)		242.0 (148.4–485.9)		151.0 (47.8–695.0)		505.6 (261.0–931.3)	
HFO	256.5 (147.5–548.0)		225.0 (109.6–395.6)		204.5 (66.4–550.0)		202.5 (17.9–348.0)	
**Cer(DiHy)16:0**		0.71		0.64		0.87		0.22
CMV	32.2 (9.3–83.1)		33.1 (16.5–71.0)		20.1 (8.3–78.3)		30.6 (20.0–175.5)	
HFO	38.3 (16.5–87.6)		30.8 (11.7–78.5)		27.5 (4.6–62.0)		24.2 (2.5–32.5)	
**Cer 18:0**		0.92		0.06		0.65		0.07
CMV	86.3 (32.3–244.8)		120.0 (59.1–183.6)		50.0 (15.5–268.0)		253.4 (101.4–392.6)	
HFO	81.5 (44.9–189.0)		73.3 (37.5–145.3)		82.5 (19.9–192.5)		94.0 (5.4–195.0)	
**Cer 18:1**		0.93		0.77		0.91		0.005
CMV	1.0 (0.5–2.6)		0.8 (0.6–2.2)		0.7 (0.1–3.4)		1.4 (1.1–4.3)	
HFO	0.9 (0.3–3.4)		1.0 (0.1–2.1)		0.8 (0.2–3.0)		0.5 (0.0–1.1)	
**Cer(DiHy)18:0**		0.36		0.29		0.72		0.08
CMV	6.0 (2.3–22.5)		16.9 (7.0–31.9)		5.0 (2.2–26.3)		22.0 (9.4–38.2)	
HFO	9.8 (4.1–29.4)		12.7 (7.2–20.0)		9.6 (2.2–27.6)		9.2 (1.0–18.8)	
**Cer(DiHy)18:1**		0.72		0.89		0.58		0.05
CMV	0.0 (0.0–0.0)		0.0 (0.0–0.0)		0.0 (0.0–1.2)		0.1 (0.0–1.2)	
HFO	0.03 (0.03–0.03)		0.03 (0.03–0.03)		0.03 (0.03–0.55)		9.2 (3.7–16.3)	
**Cer 20:0**		0.83		0.26		0.62		0.11
CMV	33.3 (12.2–89.6)		38.8 (19.3–56.1)		17.3 (4.7–94.5)		85.7 (35.9–144.6)	
HFO	31.6 (20.0–81.4)		27.7 (12.9–54.5)		31.2 (7.6–66.5)		31.8 (1.7–69.0)	
**Cer 22:0**		0.80		0.81		0.68		0.04
CMV	46.0 (15.3–98.1)		41.8 (19.5–91.6)		25.3 (6.2–137.5)		79.1 (33.8–164.6)	
HFO	33.3 (20.9–108.5)		47.5 (14.6–86.3)		59.0 (10.5–108.5)		22.0 (1.9–55.0)	
**Cer 24:0**		0.65		0.95		0.64		0.008
CMV	69.0 (20.9–132.5)		51.5 (18.1–114.4)		31.1 (5.7–217.5)		81.0 (33.6–205.2)	
HFO	46.3 (26.5–132.0)		54.2 (16.3–154.1)		53.8 (8.3–125.5)		25.3 (2.9–53.0)	
**Cer 24:1**		0.95		0.81		0.67		0.02
CMV	87.8 (28.0–201.9)		80.5 (31.6–177.9)		51.5 (10.7–397.0)		247.0 (89.0–437.8)	
HFO	65.5 (40.9–172.0)		80.5 (27.6–190.8)		110.0 (14.2–203.5)		48.3 (5.8–139.5)	
**Cer(DiHy)24:0**		0.63		0.97		0.97		0.06
CMV	6.2 (2.5–18.0)		4.1 (1.6–8.0)		2.6 (0.8–10.7)		7.1 (1.4–13.9)	
HFO	4.8 (2.7–7.7)		3.9 (1.3–11.2)		3.1 (0.3–13.6)		1.1 (1.0–5.1)	
**Cer(DiHy)24:1**		0.85		0.91		0.67		0.05
CMV	9.3 (1.6–17.2)		8.6 (3.4–15.5)		7.1 (3.5–25.9)		11.6 (3.3–42.7)	
HFO	4.7 (1.9–16.6)		9.9 (2.2–19.9)		5.7 (0.8–25.9)		3.8 (0.0–9.5)	
**SM12:0**		0.14		0.40		0.95		0.11
CMV	2.7 (1.4–5.4)		1.2 (0.6–2.4)		0.9 (0.3–2.2)		1.2 (0.6–2.7)	
HFO	2.0 (1.0–2.8)		1.0 (0.4–2.1)		1.2 (0.3–2.2)		0.6 (0.1–1.2)	
**SM(DiHy)12:0**		0.67		0.68		0.41		0.22
CMV	0.4 (0.3–1.1)		0.3 (0.2–0.6)		0.2 (0.1–0.4)		0.3 (0.2–1.4)	
HFO	0.4 (0.3–0.8)		0.3 (0.2–0.7)		0.3 (0.1–0.8)		0.2 (0.1–0.7)	
**SM16:0**		0.84		0.69		0.40		0.34
CMV	555.0 (316.3–1367.5)		808.5 (517.5–1207.5)		600.0 (173.0–1385.0)		1115.0 (697.5–1685.0)	
HFO	760.0 (348.5–1220.0)		908.3 (378.4–1733.8)		1290.0 (230.5–1862.0)		955.0 (260.0–1670.0)	
**SM18:0**		0.66		0.45		0.44		0.12
CMV	100.5 (54.2–297.6)		238.5 (123.4–373.5)		134.0 (36.5–325.0)		279.5 (150.3–512.5)	
HFO	144.5 (83.5–242.0)		197.4 (80.4–305.1)		223.5 (42.2–360.0)		169.0 (18.5–231.0)	
**SM18:1**		0.72		0.39		0.84		0.09
CMV	28.8 (14.3–96.0)		41.9 (18.4–70.4)		19.0 (5.9–82.1)		30.6 (14.8–57.6)	
HFO	26.6 (14.2–63.0)		38.6 (11.4–87.9)		36.5 (5.4–67.2)		16.0 (2.8–31.9)	
**SM24:0**		0.88		0.38		0.68		0.07
CMV	244.0 (103.4–878.8)		494.5 (241.6–707.5)		325.5 (79.5–1020.0)		515.0 (339.8–796.3)	
HFO	304.5 (128.0–550.0)		410.5 (201.7–630.0)		575.0 (88.0–982.0)		321.0 (40.5–453.0)	
**SM24:1**		0.70		0.59		0.58		0.05
CMV	178.8 (83.5–890.0)		445.8 (219.3–690.0)		299.0 (76.0–1025.0)		885.0 (444.8–1472.5)	
HFO	245.5 (115.0–419.5)		377.3 (142.6–880.0)		605.0 (96.0–1036.0)		380.0 (100.0–775.0)	

Sphingolipids were expressed as ng/ mL. Abbreviations: IQR: interquartile range; Cer: ceramide; SM: sphingomyelin. The threshold for significance was set at 0.0125.

## Discussion

In this prospective international multicenter study, we determined sphingolipid levels in tracheal aspirates of antenatally diagnosed CDH patients in the neonatal period. We found no significant differences in temporal sphingolipid profiles between patients who died/developed CLD compared to patients who survived/did not develop CLD. Furthermore, no significant sphingolipid differences were found between patients initially ventilated by CMV compared to patients initially ventilated by HFO except for ceramide-C18:1 and ceramide-C24:0 at day 14.

Bioactive sphingolipids have been investigated regarding their role in respiratory diseases such as asthma [15] and COPD [16]. Moreover, sphingolipids are important factors in lung development and disease [9], and recently have been shown to play a role in the pathogenesis of CLD in mice [17].

In lungs of preterm infants who were ventilated or received oxygen treatment, epithelial cell apoptosis and proliferation of epithelial, endothelial and smooth muscle cells were observed [18]. Various sphingolipids play an important role in cellular homeostasis. Ceramide leads to cell-cycle arrest and apoptosis, whereas sphingosine-1-phosphate is involved in facilitating proliferation and differentiation of cells. The interplay between pro-apoptotic and apoptotic ‘rheostat’ determined the formation of lung structure, both macroscopically and on a cellular level, during all stages of lung development. Since we did not find any significant difference in sphingolipid levels in tracheal aspirates of CDH patients, it seems that the role of sphingolipids in the pathophysiology of CLD is different in CDH patients when compared to for instance premature born neonates. The finding that lungs of fetuses with CDH are not surfactant deficient [7] supports this idea. No beneficial effect of surfactant replacement therapy has been shown in term neonates with CDH [8], nor in CDH neonates on ECMO [19]. Even in prematurely born neonates with CDH, surfactant replacement therapy did not improve survival rates [20]. The predisposing risk factors for CLD also vary between neonates with CDH and preterm born neonates; for example, chorioamnionitis being associated with premature birth and an increased risk of developing BPD [21] is absent in CDH. Chorioamnionitis is an inflammatory process and sphingolipids being involved in the regulation of inflammation [12], underscore a role for sphingolipids in the pathophysiology of CLD in premature infants.

Another possibility is that the lung hypoplasia seen in CDH leads to less production of ceramides essential for lung development. To solve this problem other causes of pulmonary hypoplasia should be investigated such as obstructive uropathy, but these data are neither available in our biobank nor in the literature. Besides data obtained from patients with other causes of lung hypoplasia, animal models of lung hypoplasia may be informative. Zimmer et al studied nitrofen-induced CDH rats, and performed QRT-PCR, western blotting and confocal-immunofluorescence microscopy to reveal pulmonary gene and protein expression levels of sphingosine kinase 1, sphingosine-1-phosphate 1, 2, 3 and Ras-related C3 botulinum toxin substrate 1 (Rac1) [22]. They found that pulmonary gene expression of sphingosine-1-phosphate 1 and Rac1 was significantly increased in the CDH group compared to controls, whereas sphingosine-1-phosphate 2 and 3 expression was decreased. Therefore, they concluded that sphingosine-1-phosphate 1 and Rac1 are important mediators of pulmonary hypoplasia in this model. Therefore, these factors should be included in future studies in CDH patients.

Fetal lung development occurs in a relative hypoxic environment that stimulates vascular development *via* Hypoxia Inducible Factors (HIFs) [23]. HIFs upregulate genes necessary for proper lung vascular and alveolar development [24]. Most deaths among CDH patients are due to severe pulmonary hypertension. It was recently shown that ceramide upregulation was associated with decreased Vascular Endothelial Growth Factor (VEGF) expression via suppression of HIF-1α, suggesting a role for sphingolipids in VEGF regulation [25]. Since VEGF has an important role in pulmonary vascular development, and it was shown that increased plasma VEGF-A correlates with clinical severity of pulmonary vascular disease in CDH infants [26], this mechanism could be involved in CDH patients. Our study design, however, did not provide for measuring VEGF plasma levels to test this hypothesis.

When we compared tracheal aspirate sphingolipids levels for the initial ventilation strategy, HFO versus CMV, we found no differences between the two ventilation modalities at day 1, 3 and 7. However, at day 14, two long chain ceramides (ceramide-C18:1 and ceramide-C24:0) were significantly elevated in patients initially ventilated by CMV when compared to HFO. It is remarkable that only at day 14 significant differences were observed. One explanation may be that in CMV the use of high peak inspiratory pressures and shear stress over time lead to sphingomyelinase activation and sphingomyelin degradation to ceramides. Alternatively, it is plausible that CMV increases over time *de novo* ceramide synthesis. Unfortunately, the study design did not allow inclusion of a non-ventilated control group or healthy pulmonary controls and, therefore, we do not know whether ceramide levels were increased by either ventilation modality. Ceramides enhance apoptosis and decrease vascular barrier integrity [27]. Of note, increased apoptosis has been found in epithelial cells of CLD patients, but whether this was triggered by ceramides was not investigated [28]. In the current study, the increase in very long-chain (C24:0) ceramides, known to stimulate cell proliferation and not apoptosis [29], fits with the favorable clinical outcomes in the CMV group when compared to HFO [4].

To our knowledge this is the first study investigating lung sphingolipid metabolism in CDH patients. Unique is the prospective multicenter design in a relatively large cohort of patients. Secondly, apart from the initial ventilation strategy, all children were treated according to the same study protocol [3]. A few limitations should be considered. Firstly, patient characteristics between patients of whom samples were collected and patients of whom no samples were collected, were different with respect to the side of the defect, although a recent multicenter study found that morbidity following repair of right-sided CDH was not significantly different from that in left-sided CDH survivors [30]. Therefore we believe that there was no bias in patient inclusion. Secondly, we have corrected for multiple testing for multiple time-points, but no formal testing for multiple testing of different sphingolipids was performed, which could be seen as a possible limitation. The number of TA samples decreases over time which may lead to a selection bias. This decrease is explained by the fact that some CDH infants have died and other infants were not invasively ventilated anymore. However, due to the study design TA sampling was only performed in ventilated infants. Our data were collected in a randomized clinical trial and the difference in ventilation strategy could be seen as a study limitation. However, in the multivariate analysis we adjusted for the possible effect of ventilation strategy. Third, we could only study tracheal aspirates but no lung biopsies were taken to examine gene and protein levels of the sphingolipid pathway. Therefore, future studies should also include lung biopsies to study those aspects. Since the sphingolipid pathway is quite complex and consists of more components, such as sphingosine, sphingosine 1-phosphate (S1P) and its receptors. Those components were not investigated in the current study, but have been shown in animal studies to be involved in inflammation and lung development [22]. Therefore, future studies may be developed to further study the sphingolipid pathway in more detail, including its downstream targets such as VEGF, sphingosine, S1P and its receptors. Differences between CDH infants with CDH compared to CDH infants without CLD should be studied.

In conclusion, we could not detect significant difference in temporal sphingolipid levels antenatally diagnosed CDH infants who survived/ did not develop CLD versus nonsurvivors/ survivors who developed CLD.

## Supporting Information

S1 File Minimal dataset. (SAV)

S2 File Protocol. Protocol of the randomized clinical trial on initial ventilation strategy (VICI-trial).
(PDF)

S3 File Quality report Fig 1. (PDF)
