# Supplementary material for: Sphingolipids in Congenital Diaphragmatic Hernia; Results from an International Multicenter Study
Source: PLoS One. 2016 May 9;11(5):e0155136. doi: 10.1371/journal.pone.0155136 (PMC4861280; doi:10.1371/journal.pone.0155136)
Supplement: S2 File — Protocol of the randomized clinical trial on initial ventilation strategy (VICI-trial). (PDF) [file pone.0155136.s002.pdf]

Conventional Mechanical Ventilation Versus High-frequency Oscillatory Ventilation for Congenital Diaphragmatic Hernia

A Randomized Clinical Trial (The VICI-trial)

Kitty G. Snoek, MD, Irma Capolupo, MD, PhD,† Joost van Rosmalen, PhD,‡ Lieke de Jongste-van den Hout, MD, PhD,* Sanne Vijfhuize, MD,* Anne Greenough, MD,§ René M. Wijnen, MD, PhD,* Dick Tibboel, MD, PhD,* and Irwin K.M. Reiss, MD, PhD*, CDH EURO Consortium*

Objectives: To determine the optimal initial ventilation mode in congenital diaphragmatic hernia.

Background: Congenital diaphragmatic hernia is a life-threatening anomaly with significant mortality and morbidity. The maldeveloped lungs have a high susceptibility for oxygen and ventilation damage resulting in a high incidence of bronchopulmonary dysplasia (BPD) and chronic respiratory morbidity.

Methods: An international, multicenter study (NTR 1310), the VICI-trial was performed in prenatally diagnosed congenital diaphragmatic hernia infants (n = 171) born between November 2008 and December 2013, who were randomized for initial ventilation strategy.

Results: Ninety-one (53.2%) patients initially received conventional mechanical ventilation and 80 (46.8%) high-frequency oscillation. Forty-one patients (45.1%) randomized to conventional mechanical ventilation died/had BPD compared with 43 patients (53.8%) in the high-frequency oscillation group. An odds ratio of 0.62 [95% confidence interval (95% CI) 0.25–1.55] ($P = 0.31$) for death/BPD for conventional mechanical ventilation vs high-frequency oscillation was demonstrated, after adjustment for center, head-lung ratio, side of the defect, and liver position. Patients initially ventilated by conventional mechanical ventilation were ventilated for fewer days ($P = 0.03$), less often needed extracorporeal membrane oxygenation support ($P = 0.007$), inhaled nitric oxide ($P = 0.045$), sildenafil ($P = 0.004$), had a shorter duration of vasoactive drugs ($P = 0.02$), and less often failed treatment ($P = 0.01$) as compared with infants initially ventilated by high-frequency oscillation.

Conclusions: Our results show no statistically significant difference in the combined outcome of mortality or BPD between the 2 ventilation groups in prenatally diagnosed congenital diaphragmatic hernia infants. Other outcomes, including shorter ventilation time and lesser need of extracorporeal membrane oxygenation, favored conventional ventilation.

From the *Intensive Care and Department of Pediatric Surgery, Erasmus Medical Centre-Sophia Children's Hospital, Rotterdam, The Netherlands; †Department of Medical and Surgical Neonatology, Bambino Gesù Hospital, Rome, Italy; ‡Department of Biostatistics, Erasmus Medical Centre, Rotterdam, The Netherlands; and §Division of Asthma, Allergy and Lung Biology, MRC & Asthma UK Centre in Allergic Mechanisms of Asthma, King's College London, London, UK.

CDH EURO Consortium; Alphabetic order of institution of the participating investigators (provided and cared for study patients): Irma Capolupo, Department of Medical and Surgical Neonatology, Bambino Gesù Hospital; Alessandra di Pede, Department of Medical and Surgical Neonatology, Bambino Gesù Hospital; Andrea Dotta, Department of Medical and Surgical Neonatology, Bambino Gesù Hospital; Pietro Bagolan, Department of Medical and Surgical Neonatology, Bambino Gesù Hospital; Kitty G. Snoek, Intensive Care and Department of Pediatric Surgery, Erasmus Medical Centre-Sophia Children's Hospital; Lieke de Jongste-van den Hout, Intensive Care and Department of Pediatric Surgery, Erasmus Medical Centre-Sophia Children's Hospital; Sanne Vijfhuize, Intensive Care and Department of Pediatric Surgery, Erasmus Medical Centre-Sophia Children's Hospital; Ulrike Kraemer, Intensive Care and Department of Pediatric Surgery, Erasmus Medical Centre-Sophia Children's Hospital; Irwin K.M. Reiss, Intensive Care and Department of Pediatric Surgery, Erasmus Medical Centre-Sophia Children's Hospital; Dick Tibboel, Intensive Care and Department of Pediatric Surgery, Erasmus Medical Centre-Sophia Children's Hospital; René M. Wijnen, Intensive Care and Department of Pediatric Surgery, Erasmus Medical Centre-Sophia Children's Hospital; Carla Pinto, Department of Pediatric Intensive Care, Hospital Pediatrico de Coimbra; Maria Gorett Silva, Department of Pediatrics, Division of Neonatology, Hospital Sao Joao; Joana Saldanha, Department of Pediatrics, Hospital de Santa Maria; Prashanth Bhat, Division of Asthma, Allergy and Lung Biology, MRC & Asthma UK Centre in Allergic Mechanisms of Asthma, King's College London; Vadivelam Murthy, Division of Asthma, Allergy and Lung Biology, MRC & Asthma UK Centre in Allergic Mechanisms of Asthma, King's College London; Anne Greenough, King's College London; Arno van Heijst, Department of Neonatology, Radboudumc; Thomas Schaible, Department of Pediatrics, Universitätsklinikum Mannheim; Lucas Wessel, Department of Pediatric Surgery, Universitätsklinikum Mannheim; Karel Allegaert, Department of Neonatology, Universiteitsziekenhuis Gasthuisberg; Anne Debeer, Department of Neonatology, Universiteitsziekenhuis Gasthuisberg.

Declarations: This research was partly funded by SSWO, Willem H. Kröger foundation. Dr. Greenough has held grants from various ventilator manufacturers and received honoraria for giving lectures and advising various ventilator manufacturers. The other authors declare no conflicts of interest.

Reprints will not be available from the authors.

Reiss had full access to all the data in the study and had final responsibility for the decision to submit for publication. Van Rosmalen, Snoek, and Reiss conducted the analyses and are responsible for the data analyses.

Capolupo, de Jongste-van den Hout, Greenough, Wijnen, Tibboel, Reiss did the study concept and design.

Snoek, Capolupo, de Jongste- van den Hout, Vijfhuize, Greenough, Wijnen, Tibboel, Reiss, di Pede, Dotta, Bagolan, Kraemer, Pinto, Gorett, Saldanha, Bhat, Murthy, van Heijst, Schaible, Wessel, Allegaert, Debeer did the acquisition, analysis, or interpretation of data.

Snoek, van Rosmalen, Greenough, Tibboel, Reiss did the drafting of manuscript.

Snoek, Capolupo, van Rosmalen, de Jongste- van den Hout, Vijfhuize, Greenough, Wijnen, Tibboel, Reiss, di Pede, Dotta, Bagolan, Kraemer, Pinto, Gorett, Saldanha, Bhat, Murthy, van Heijst, Schaible, Wessel, Allegaert, Debeer did the critical revision of the manuscript for important intellectual content.

Tibboel, Wijnen, Reiss did the administrative, technical, or material support.

Tibboel, Reiss did the supervision of the study.

Supplemental digital content is available for this article. Direct URL citations appear in the printed text and are provided in the HTML and PDF versions of this article on the journal's Web site (www.annalsofsurgery.com).

Reprints: Irwin K.M. Reiss, MD, PhD, Intensive Care and Department of Neonatology, Erasmus Medical Centre- Sophia Children's Hospital, Room Sp-4459, P.O. Box 3060, NL-3000 CB Rotterdam, The Netherlands. E-mail: i.reiss@erasmusmc.nl

Copyright © 2015 Wolters Kluwer Health, Inc. All rights reserved.

ISSN: 0003-4932/14/26105-0821
DOI: 10.1097/SLA.0000000000001533

Copyright © 2015 Wolters Kluwer Health, Inc. Unauthorized reproduction of this article is prohibited.

Keywords: congenital diaphragmatic hernia, conventional mechanical ventilation, high frequency oscillation

(*Ann Surg* 2015;xx:xxx–xxx)

Congenital diaphragmatic hernia (CDH) is a developmental defect of the diaphragm that occurs in approximately 1 in 3000 live births.[1] It is characterized by maldevelopment of both the ipsilateral and contralateral lung and abnormal prenatal pulmonary vascular growth. Although pulmonary hypoplasia and pulmonary hypertension are the main causes of mortality, ventilator-induced lung injury and oxygen toxicity may result in prolonged oxygen dependency, or bronchopulmonary dysplasia (BPD).[2] Logan et al[3] have suggested that 25% of the mortality is due to potentially treatable aspects of the underlying pathophysiology and that ventilator-induced lung injury could be one of these aspects.

Since 2008, all CDH patients born in European countries represented in the CDH EURO consortium have been treated according to a standardized neonatal treatment protocol that was developed at a consensus meeting.[4] After implementation of this protocol, mortality decreased from 33% to 12%.[5] This decrease in mortality rate should be interpreted against of the year upon year variability in mortality rates. Nevertheless, standardization of care is an ideal backcloth to undertake multicenter randomized controlled trials (RCTs).

The optimal initial invasive ventilation strategy in antenatal diagnosed CDH patients, however, is still unknown. Certain studies with retrospective or observational study designs have shown improved survival and lower incidence of BPD with the use of high-frequency oscillation (HFO) ventilation.[6–11] They reported that HFO may result in favorable outcome in CDH because of better oxygenation and higher mean airway pressure without increasing the incidence of barotrauma. In another study, however, based on the CDH EURO consortium registry, HFO as initial ventilation mode was associated with increased rates of mortality and BPD.[2] We, therefore, performed the first prospective multicenter study (VICI-trial) of conventional mechanical ventilation (CMV) or HFO as the initial ventilation strategy.[12] The primary outcome measure was death or BPD.[13]

METHODS

A prospective, randomized clinical trial was performed. All participating centers were members of the CDH EURO consortium.[4] Prenatally diagnosed CDH infants, born at a gestational age of more than 34 weeks between November 2008 and December 2013 in 1 of the 9 centers, were eligible for inclusion. Exclusion criteria were severe chromosomal anomaly such as trisomy 13 or 18, which may imply a decision to stop or not to start medical treatment; severe cardiac anomalies expected to need corrective surgery in the first 60 days after birth; renal anomalies associated with oligohydramnios; severe orthopedic and skeletal deformities that were likely to influence thoracic or lung development; and severe anomalies of the central nervous system. We excluded patients with a gestational age of less than 34 weeks so that the results could not be influenced by lung prematurity. Besides, neonates with a gestational age below 34 weeks cannot be placed on extracoporeal membrane oxygenation (ECMO). Ethical approval was given by the medical ethics review board of Erasmus MC, Rotterdam, the Netherlands (NTR 1310). Thereafter, all local medical ethical committees gave their approval. Parents gave written informed consent. The procedures, including obtaining informed consent, were conducted in accord with the ethical standards of the Committee on Human Experimentation of the institution in which the experiments were done. The centers' duration of experience with HFO before initiation of the VICI-trial ranged from 8 to 19 years.

To achieve equal distribution of the 2 ventilation modes among the participants, block randomization stratified per center was carried out using a computer-generated randomization schedule for each center by a 24-hour interactive web response system. After birth, infants were centrally randomized to either CMV or HFO. The allocated ventilation mode was started within 2 hours after birth. Patients were monitored up to 1 year of life or until discharge whichever came first. CMV was provided by a neonatal ventilator capable of positive pressure ventilation or triggered modes. Initial settings were a positive inspiratory pressure (PIP) of 20 to 25 cmH_2O and a positive end-expiratory pressure (PEEP) of 3 to 5 cmH_2O, with a ventilator rate of 40 to 60/min. According to clinical practice, PIP was increased in the case of ventilation problems and the PEEP or FiO_2 were adjusted if oxygenation problems occurred. Weaning from ventilation was preferentially by means of decreasing PIP or frequency to achieve $PaCO_2$ levels above 45 mm Hg. HFO was provided by a high-frequency oscillatory ventilator. Initial settings were mean airway pressure 13 to 17 cmH_2O, frequency 10 to 12 Hz, delta P 30 to 50 cmH_2O depending on chest wall vibration. According to clinical practice, the frequency was reduced and/or delta P was increased in the case of ventilation problems and the MAP and/or FiO_2 were adjusted if oxygenation problems occurred. All patients were treated according to the same standardized CDH EURO Consortium neonatal treatment protocol.[4] The initially allocated ventilation mode could be switched if one or more of the following predetermined failure criteria were met at 2 consecutive time points for at least 3 hours: inability to maintain preductal saturations above 85% ($\pm$52 mm Hg or 7 kPa) or postductal saturations above 70% ($\pm$5.3 kPa or 40 mm Hg); increase in $CO_2 > 65$ mm Hg or 8.5 kPa despite optimization of ventilatory management; PIP > 28 cmH_2O; mean airway pressure >17 cmH_2O; inadequate oxygen delivery with metabolic acidosis defined as lactate ≥ 5 mmol/L and pH < 7.20; hypotension resistant to fluid therapy and inotropic support resulting in a urine output <0.5 ml/kg/hour; oxygenation index of longitudinal evaluation ≥ 40. Patients could experience one or more criteria for treatment failures. If a patient born in an ECMO center should meet one of these failure criteria, either an ECMO procedure was considered to be initiated or the initially allocated ventilation mode was considered to be switched. If subsequently there was no improvement, the infant was placed on ECMO. If a patient born in a center without ECMO availability should meet one of these failure criteria, only the ventilation mode was considered to be switched. None of the patients was transferred from a non-ECMO center to an ECMO-center. Arterial blood pressure was to be maintained at a normal level for gestational age. In case of hypotension and/or poor perfusion, 10 to 20 ml/kg NaCl 0.9% was to be administered 1 to 2 times and inotropic agents were considered according to the local practice. If there was suprasystemic pulmonary artery pressure and right-to-left shunting through the foramen ovale, administration of intravenous phosphodiesterase type-5 inhibitor E5 (PDE-5) (Sildenafil) was to be considered. According to the protocol, paralysis in the delivery room was to be avoided if possible. Antenatal data, neonatal characteristics as well as data on the clinical course, and treatment were collected. Defect size was classified intraoperatively according to the CDH study group.[14] Liver position was determined during surgery or if there was no surgical repair from prenatal echographic data. Pulmonary hypertension was categorized as none, <2/3 systemic, 2/3 systemic to systemic, or suprasystemic. The latter 3 categories are according to the definition of Keller et al.[15]

Sample Size Calculation

In each ventilation category, 187 infants were to be randomized to give 80% power using a 5% significance level to detect a 15% difference in the combined primary outcome of death or BPD. BPD

© 2015 Wolters Kluwer Health, Inc. All rights reserved.

Copyright © 2015 Wolters Kluwer Health, Inc. Unauthorized reproduction of this article is prohibited.

was defined as oxygen dependency beyond 28 days after birth.[13] To allow for some nonevaluable patients and dropouts, inclusion of 200 patients per group was planned.

Statistical Analysis

To determine whether differences in the demographics of the 2 treatment arms were statistically significant, independent samples *t* tests for continuous variables that were normally distributed, χ^2 tests for categorical variables, and Mann-Whitney *U* tests for continuous variables that were not normally distributed, were used. Multiple logistic regression analysis using treatment arm, center, lung-to-head ratio, position of the liver, and side of the defect as independent variables served to evaluate the primary outcome. A subgroup analysis was performed for infants who underwent surgical repair, with defect size added to the independent variables to evaluate the primary outcome. The subgroup analysis and the independent variables for the logistic regression model were selected a priori. However, the 4 centers that each treated fewer than 10 patients in total, were evaluated as a single center in all analyses. Missing data of lung-to-head ratio ($n = 23$ patients) and diaphragmatic defect size ($n = 5$) were imputed by automatic multiple imputation in SPSS with 100 imputations. The predictors in the imputation models consisted of BPD or death, as well as all independent variables (except initial ventilation mode) in the logistic regression models. The goodness-of-fit of the logistic regression models was assessed using the Hosmer-Lemeshow test. Patients were analyzed in the group of randomization, even after switching of ventilator mode. Overall mortality within first 60 days of life was calculated using Kaplan-Meier curves and compared between treatment arms using a log-rank test stratified by center. The Mantel-Haenszel test with stratification by center was used to compare overall mortality in the first year of life, presence of pulmonary hypertension, iNO, vasoactive medication, phosphodiesterase inhibitor 5 (Sildenafil, Pfizer, Basel, Switzerland), and requirement of ECMO (in ECMO centers only) between treatment arms. The Van Elteren test with stratification by center was used to compare the difference of severity of BPD, number of days of ventilation, the number of treatment failures, severity of pulmonary hypertension, and number of days treated with vasoactive medication between the 2 treatment arms. Treatment failures were only recorded (ie, those infants whose initial ventilation mode was switched or who were placed on ECMO) for patients whose ventilator mode was switched and those who were placed on ECMO. All statistical tests were 2-sided, and a *P* value of <0.05 was considered statistically significant. The analyses were conducted using IBM SPSS 21.0 for Windows (IBM Corp., Armonk, NY) for the primary outcome and STATA 13.0 for Windows (StataCorp LP, College Station, TX) for secondary outcomes.

RESULTS

Between November 2008 and December 2013, 171 antenatally diagnosed CDH patients from 9 European centers were included (Fig. 1). The predicted severity of illness (SNAP-II score and O/E LHR) did not significantly differ between the 2 groups (Table 1). The characteristics of the 448 nonincluded patients were not significantly different for the prevalence of death as compared with the included patients (Table 2). None of the included patients was withdrawn from the study and the primary outcome was observed for all included patients. ECMO was available in 6 of the 9 centers (involving 100 patients), in 2 centers it was not (17 patients), and in 1 center ECMO was introduced in 2013 (46 patients before ECMO and 8 patients after ECMO). Forty of the 108 (37%) patients born in a center with ECMO availability were subsequently supported by ECMO (Fig. 2). Although the protocol dictated that the allocated ventilator mode should be started within 2 hours after birth, in all children, it was started within 1 hour after delivery.

Forty-one of the 91 patients (45.1%) initially ventilated with CMV died or had BPD by day 28 compared with 43 of the 80 patients

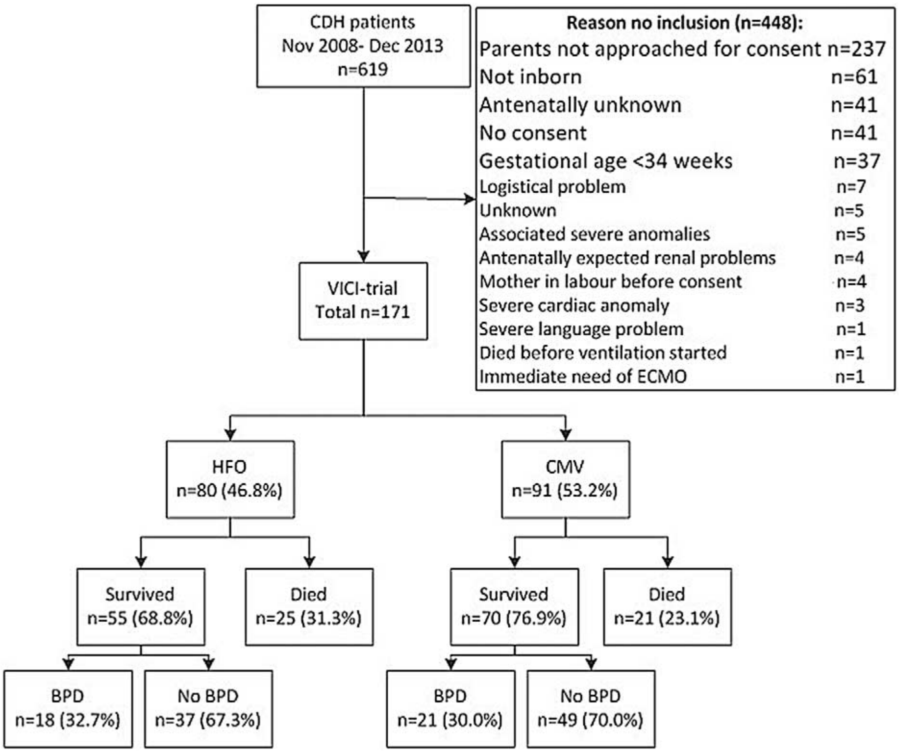

FIGURE 1. Flowchart of patient inclusion. Explanatory legend. BPD indicates bronchopulmonary dysplasia; CDH, congenital diaphragmatic hernia; CMV, conventional mechanical ventilation; ECMO, extracorporeal membrane oxygenation; HFO, high-frequency oscillation.

© 2015 Wolters Kluwer Health, Inc. All rights reserved.

Copyright © 2015 Wolters Kluwer Health, Inc. Unauthorized reproduction of this article is prohibited.

TABLE 1. Baseline Characteristics by Randomized Ventilation Mode

Variable	HFO (n = 80)	CMV (n = 91)	*P*
FETO	12 (15.0%)	7 (7.7%)	0.15
LHR	1.51 (1.07–1.92)	1.53 (1.13–2.00)	0.67
O/E LHR	47% (15%–141%)	48% (21%–100%)	0.46
Gestational age	38 (37.3–39.0)	38.1 (37.4–38.9)	0.39
Birth weight (kg)	2.89 (0.47)	2.95 (0.46)	0.38
Male sex	36 (45.0%)	48 (52.7%)	0.36
SNAP-II score	25.0 (14.0–40.0)	21.0 (10.0–40.0)	0.44
Left side CDH	73 (91.3%)	75 (82.4%)	0.12
Liver			0.76
Intrathoracic	46 (57.5%)	55 (60.4%)	
Abdominal	34 (42.5%)	36 (39.6%)	
Type of repair			0.73
Primary closure	26 (32.5%)	27 (29.7%)	
Patch repair	42 (52.5%)	50 (54.9%)	
No repair	12 (15.0%)	14 (15.4%)	
Diaphragmatic defect size			0.10
A	6 (7.5%)	5 (5.5%)	
B	21 (26.3%)	28 (30.8%)	
C	28 (35.0%)	40 (44.0%)	
D	10 (12.5%)	2 (2.2%)	
No repair	12 (15.0%)	14 (15.4%)	
Unknown	3 (3.8%)	2 (2.2%)	
Major cardiac anomaly			0.42
Aortic hypoplasia	2 (2.6%)	0 (0%)	
ASD and VSD	1 (1.3%)	0 (0%)	
HLHS variant	0 (0%)	1 (1.1%)	
Aortic stenosis	0 (0%)	1 (1.1%)	
No cardiac anomaly	77 (96.3%)	89 (97.8%)	
Age at repair (days)	5.0 (3.0–9.0)	4.0 (3.0–5.0)	0.005
Centers			0.43
1: (ECMO)	19 (46.3%)	22 (53.7%)	
2: (ECMO)	7 (41.2%)	10 (53.8%)	
3: (no ECMO)	7 (43.8%)	9 (56.2%)	
4: (ECMO since 01–01–2013)	30 (55.6%)	24 (44.4%)	
5: (ECMO)	12 (48.0%)	13 (52.0%)	
6: (ECMO)	3 (37.5%)	5 (62.5%)	
7: (ECMO)	1 (12.5%)	7 (87.5%)	
8: (no ECMO)	1 (100%)	0 (0%)	
9: (ECMO)	0 (0%)	1 (100%)	

Results presented as n (%), mean (SD), or median (IQR). ASD, atrial septal defect; FETO, fetoscopic endotracheal occlusion; HLHS, hypoplastic left heart syndrome; LHR, lung-to-head ratio; O/E LHR: observed to expected lung-to-head ratio; PH, pulmonary hypertension; SNAP-II score, score for neonatal acute physiology-II; VSD, ventricular septal defect.

(53.8%) in HFO. That difference was not statistically significant after taking into account of center, lung-to-head ratio, side of the defect, and position of the liver, with an odds ratio (OR) of 0.62 [95% confidence interval (95% CI) 0.25–1.55] ($P = 0.31$) comparing CMV with HFO (Table 3). LHR (OR 0.164, $P < 0.001$, 95% CI 0.064–0.420) and liver position (OR 9.47, $P < 0.001$, 95% CI 3.402–26.359) were significantly associated with a worse outcome. The primary outcome results without pooling of the centers are shown in Supplemental Digital Content 1, http://links.lww.com/SLA/A909. A subgroup analysis of the 145 operated infants taking into account center, lung-to-head ratio, side of the defect, position of the liver, and defect size demonstrated that 27 of 77 patients (35.1%) in CMV and 31 of 68 patients (45.6%) in HFO died or had BPD by day 28; OR of 0.76 (95% CI 0.24–2.41) ($P = 0.64$) (Table 4). Median (interquartile range, IQR) values in the Hosmer-Lemeshow test in the

TABLE 2. Background Characteristics Nonparticipants

Variable	n = 448
Sex	
female	167/411 (40.6%)
Unknown	37 (8.3%)
Gestational age (weeks)	36.8 (3.0)
Birth weight (kg)	2.76 (0.68)
Side	
Left	345/421 (81.9%)
Right	73/421 (17.3%)
Bilateral	3/421 (0.7%)
Unknown	27 (6.0%)
Inborn	320/425 (75.3%)
Unknown	23 (5.1%)
Death	105/420 (25.0%)
Unknown	28 (6.3%)

Results are presented as n (%) or mean (SD).

primary outcome of all patients were 0.306 (0.178 to 0.481) and in the subgroup analyses 0.653 (0.547 to 0.779).

The duration of survival within first 60 days after birth did not differ between the 2 groups ($P = 0.19$) (Fig. 3). The overall mortality in the first year after birth was 21 patients (23.1%) in the CMV group and 25 patients (31.3%) in the HFO group ($P = 0.26$). The median duration of ventilation was 10.0 days (IQR 6.0 to 18.0 days) in the CMV group and 13.0 days (IQR 8.0 to 23.0 days) in the HFO group ($P = 0.03$) (Table 5). A subgroup analysis of the 108 patients born in a center with ECMO availability showed that 16 patients (26.2%) initially ventilated by CMV received ECMO vs 24 patients (51.1%) ventilated by HFO ($P = 0.007$). The number of treatment failures was significantly different between the 2 treatment groups, 27 patients (33.8%) in the HFO group vs 20 patients (22.0%) in the CMV group ($P = 0.01$). Of the 22 infants who were initially ventilated by CMV and due to treatment failures that switched to HFO, 14 (63.6%) died, 18 (81.8%) died or had BPD by day 28, and 8 (36.4%) also received ECMO treatment. In the CMV group, 39 patients (42.9%) received iNO vs 45 patients of the 80 patients (56.2%) in the HFO group ($P = 0.045$). Eleven patients (12.1%) initially ventilated by CMV received a phosphodiesterase 5 inhibitor vs 25 patients (31.2%) initially ventilated by HFO ($P = 0.004$). The median duration of vasoactive medication was 6.0 days (IQR 3.3 to 11.8 days) in the CMV group and 8.0 days (IQR 4.3 to 19.0 days) in HFO ($P = 0.02$). The median age at repair was 5.0 days (IQR 3.0 to 9.0 days) in the CMV group and 4.0 days (IQR 3.0–5.0 days) in the HFO group ($P = 0.005$). The median length of ICU stay of the survivors in the CMV group was 23.0 days (IQR 23.8 to 35.3 days) and that of the survivors in the HFO group 20.0 days (IQR 13.0 to 54.0 days), $P = 0.99$. Patients born in a center with ECMO facilities as compared with patients born in a center without ECMO facilities were not significantly different for the primary outcome (died or BPD), overall mortality in the first year of life, length of ventilation, severity of BPD, frequency of iNO, vasoactive medication, number of treatment failures, and switching of ventilation mode (Supplemental Digital Content 2, http://links.lww.com/SLA/A909). In Supplemental Digital Content 3a and 3b, http://links.lww.com/SLA/A909, secondary outcomes per ventilation group and differences between ECMO centers and non-ECMO centers are shown. In the sensitivity analysis, there was no significant interaction between ECMO availability and type of initial ventilation (OR 2.05, $P = 0.44$).

The trial was stopped early after enrolment of 171 participants in an inclusion period of 5 years because of lower than anticipated recruitment rates and due to a lack of financial resources and a lack of research infrastructure in 1 high-volume center.

© 2015 Wolters Kluwer Health, Inc. All rights reserved.

Copyright © 2015 Wolters Kluwer Health, Inc. Unauthorized reproduction of this article is prohibited.

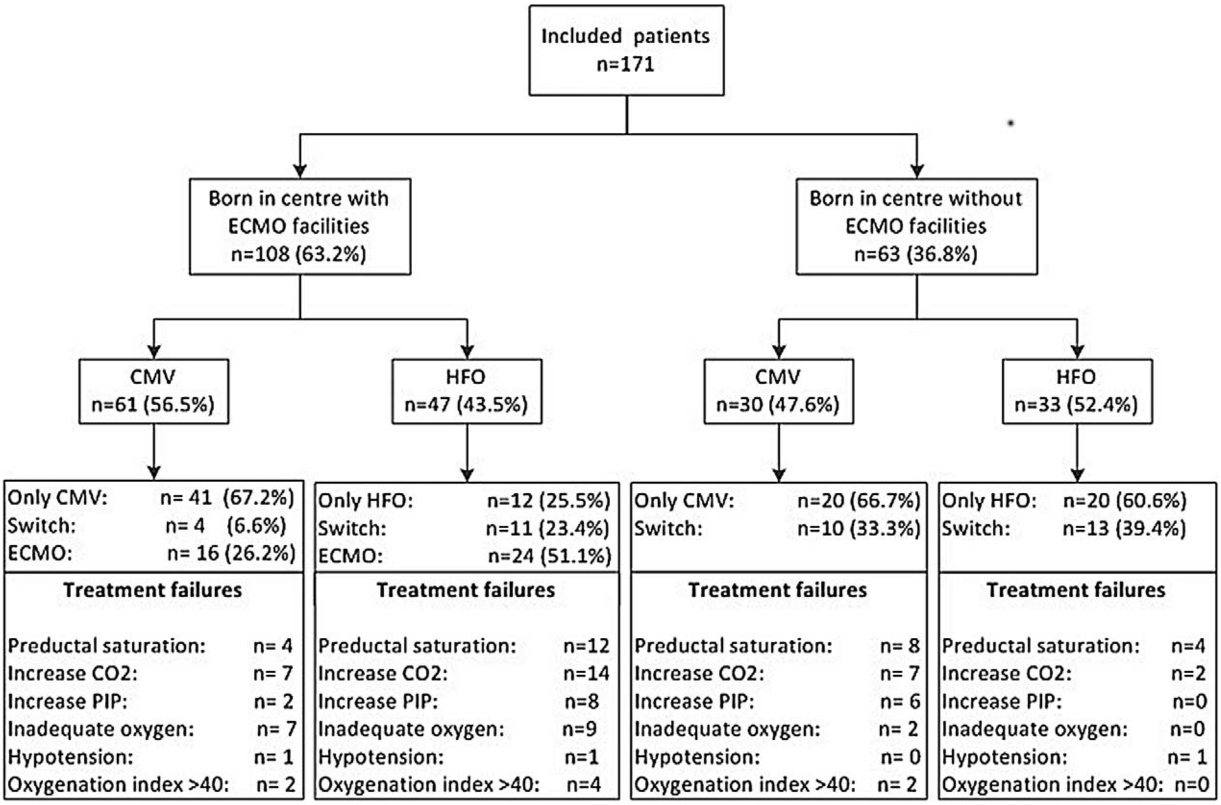

FIGURE 2. Flowchart treatment failures. Explanatory legend. CMV indicates conventional mechanical ventilation; ECMO, extracorporeal membrane oxygenation; HFO, high-frequency oscillation; PIP, peak inspiratory pressure. Preductal saturation: inability to maintain preductal saturations above 85% (+52 mm Hg or 7 kPa) or postductal saturations above 70% (±5.3 kPa or 40 mm Hg). Increase CO_2: increase in CO_2 >65 mm Hg or 8.5 kPa despite optimization of ventilatory management. Increase PIP: peak inspiratory pressure >28 cmH_2O; mean airway pressure >17 cmH_2O. Inadequate oxygen: inadequate oxygen delivery with metabolic acidosis defined as lactate ≥5 mmol/L and pH <7.20. Hypotension: hypotension resistant to fluid therapy and inotropic support resulting in a urine output <0.5 ml/kg/hour. Oxygenation index >40: oxygenation index of longitudinal evaluation ≥40.

TABLE 3. Primary Outcome: All Patients (n = 171)

Variable	OR	95% CI	*P*
Ventilation			
HFO	Ref		
CMV	0.620	0.249–1.548	0.306
LHR	0.166	0.067–0.413	0.000
Liver			
Down	Ref		
Up	10.574	4.006–27.911	0.000
Side			
Left	Ref		
Right	0.986	0.235–4.139	0.985
Centers			
Center (1)	Ref		
Center(2)	0.221	0.034–1.453	0.116
Center (3)	0.114	0.019–0.683	0.017
Center (4)	0.210	0.038–1.152	0.072
Center (5)	0.440	0.133–1.455	0.179
Center (6)	2.310	0.461–11.585	0.309

95% CI indicates 95% confidence interval; CMV, conventional mechanical ventilation; HFO, high-frequency oscillation; OR, odds ratio. Ref: reference category.

DISCUSSION

In this first RCT comparing HFO and CMV in infants prenatally diagnosed with CDH, we have demonstrated no statistically significant difference in the combined outcome of mortality or BPD between the 2 ventilation groups. The infants initially supported by CMV required a significantly shorter duration of ventilation and inotrope support, however, and were less likely to receive vasoactive medication or phosphodiesterase type 5 inhibitors or be placed on ECMO.

Previous animal studies as well as observational and retrospective human studies about the optimal mechanical ventilation strategy in CDH have shown contradictory results.[2,8,9] On the one hand, animal studies have suggested that HFO could improve pulmonary gas exchange, minimize barotrauma, and decrease the presence of inflammatory mediators.[16,17] The models used in those studies are not comparable, however, to the clinical situation, as in the animal models, the CDH was induced by interfering with development of the lungs in a normally programmed lung antenatally, which is quite different from the pathophysiology of the abnormal lung development in CDH. On the other hand, Wilson et al[18] showed in infants that there was no difference in outcome between HFO and CMV ventilation. That study, however, was

© 2015 Wolters Kluwer Health, Inc. All rights reserved.

Copyright © 2015 Wolters Kluwer Health, Inc. Unauthorized reproduction of this article is prohibited.

TABLE 4. Subgroup Analyses in Operated Patients (n = 145)

Variable	OR	95% CI	*P*
Ventilation			
HFO	Ref		
CMV	0.755	0.237–2.412	0.636
LHR	0.157	0.043–0.578	0.005
Liver			
Down	Ref		
Up	8.234	2.230–30.400	0.002
Side			
Left	Ref		
Right	0.764	0.096–6.065	0.799
Defect size			
Defect size A	Ref		
Defect size B	6.087	0.145–255.322	0.343
Defect size C	9.915	0.260–378.726	0.217
Defect size D	49.831	0.588–4222.363	0.084
Centers			
Center (1)	Ref	Ref	Ref
Center (2)	0.110	0.010–1.213	0.071
Center (3)	0.087	0.008–0.896	0.040
Center (4)	0.048	0.005–0.480	0.010
Center (5)	0.155	0.032–0.750	0.020
Center (6)	2.386	0.424–13.426	0.324

95% CI indicates 95% confidence interval; CMV, conventional mechanical ventilation; HFO, high-frequency oscillation; OR, odds ratio. Ref: reference category.

performed before the introduction of a gentle ventilation strategy with permissive hypercapnia. We found that infants initially ventilated by HFO needed mechanical ventilation for a longer time despite no significant difference in markers of severity such as SNAP-II score and O/E LHR between the 2 ventilation groups. A possible explanation is the process by which HFO causes overinflation of the terminal lung units, and especially of the ipsilateral lung, which leads to disruption of the epithelium and thereby to retained secretions and debris. Due to that process, the alveoli could possibly be more vulnerable to inflammation.[19]

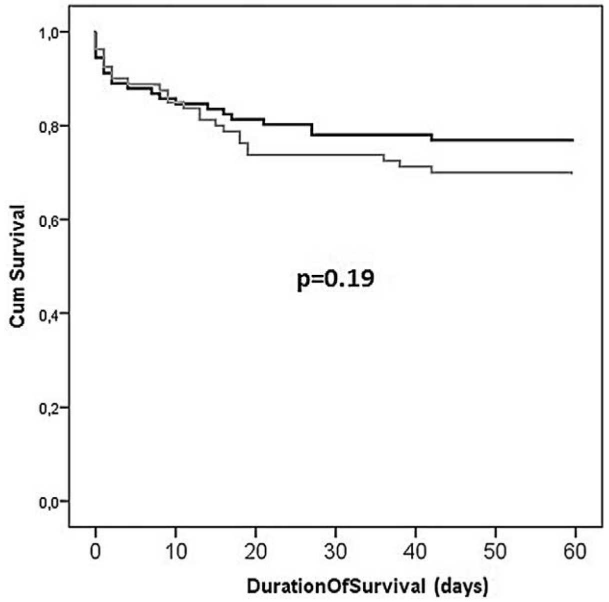

FIGURE 3. Kaplan-Meier curve of all included patients comparing CMV and HFO. Explanatory legend. Black: Conventional mechanical ventilation. Grey: High-frequency oscillation.

Moreover, the intubation and endotracheal suctioning procedures in mechanical ventilation can lead to injury of the tracheobronchial tree and—possibly even more important—to damage to the ciliated cells of the tracheal epithelium and mucociliary transport system.[19] We did not document the frequency of endotracheal suctioning and it is debatable whether this is more often needed in HFO than CMV, but the longer duration of HOFV may have been associated with more endotracheal suctioning procedures. Although in HFO tidal volumes are very low, high levels of PEEP with constant tidal volumes may also exacerbate VILI.[20] Schultz et al[21] found that prolonged mechanical ventilation induced pulmonary inflammation in preterm infants by increasing pulmonary edema. As infants with HFO were ventilated longer, this could have contributed to more pulmonary inflammation.[21] In future studies, tracheal aspirates of CDH patients examining sphingolipids levels may possibly give further insight in the exact mechanism of VILI.[22]

Infants in the HFO group received vasoactive medication for a longer period and were significantly more likely to receive iNO and a phosphodiesterase type 5 inhibitor. Those treatment modalities are used in the treatment of pulmonary hypertension.[15] We also found that infants initially ventilated by CMV were significantly less likely to receive ECMO treatment. The above results suggest that in the CMV group, the occurrence of pulmonary hypertension was less frequent and that it was less severe. The protocol dictated echocardiography only in the first 24 hours after birth, in which period we documented no significant difference in the occurrence and severity of pulmonary hypertension. However, subsequent echocardiographies were undertaken that influenced management of the patients.

Some limitations of this study should be considered. Firstly, we did not achieve the calculated sample size. Secondly, we excluded patients born before 34 weeks of gestation, as these infants could additionally have respiratory distress syndrome and the results of surfactant administration in this group are poor.[23] Moreover, very poor survival has been reported in CDH infants born before 33 weeks of gestation treated by FETO.[24] Almost 10% of the parents refused to participate in the study. Due to limited financial resources and a lack of research infrastructure in 1 high-volume center, inclusion was stopped after 1 year and 8 months. We had extended the inclusion period from 3 to 5 years and we calculated that the study would need to continue for a total period of 10 years to achieve the sample size. At that time, we had no expectation of an improving inclusion rate and saw no reason to further extend the inclusion period and therefore stopped the study. The data were not analyzed until after this decision and thus the decision was not influenced by preliminary results.

Using the assumptions of the original power calculation, the attained sample size (n = 80 for the HFO group and n = 91 for the CMV group) yields a power of at least 44% to detect a difference of 15% in primary outcome between the 2 ventilation groups. After a child's condition had stabilized situation in the delivery room, the child was transferred to the ICU and received the allocated ventilation strategy. In all children, the allocated ventilator support was started within 1 hour after birth, but it cannot be excluded that receiving the other type of ventilation in the delivery room before that has influenced our results.

The major strengths of the current study was the fact that it was carried out in as many as 9 European centers in which all CDH patients are treated according to a standardized neonatal treatment protocol.[4] All centers had at least a period of more than 10 years of experience with HFO so we do not think that the factor ''experience'' has influenced our results. Secondly, central randomization stratified per center was carried out. We have reported size of the defect, major

© 2015 Wolters Kluwer Health, Inc. All rights reserved.

Copyright © 2015 Wolters Kluwer Health, Inc. Unauthorized reproduction of this article is prohibited.

TABLE 5. Secondary Outcomes by Ventilation Group Corrected for Center

Variable	HFO (n = 80)	CMV (n = 91)	*P*
Overall mortality in first year of life	25 (31.3%)	21 (23.1%)	0.26
Length of ventilation (days)	13.0 (8.0–23.0)	10.0 (6.0–18.0)	0.03
Severity of BPD			0.13
No BPD	37 (46.3%)	50 (54.9%)	
Mild BPD	7 (8.8%)	13 (14.3%)	
Moderate BPD	2 (2.5%)	1 (1.1%)	
Severe BPD	9 (11.3%)	6 (6.6%)	
Died	25 (31.3%)	21 (23.1%)	
ECMO (in ECMO centers only)	24/47 (51.1%)	16/61 (26.2%)	0.007
Inhaled nitric oxide	45 (56.2%)	39 (42.9%)	0.045
Phosphodiesterase inhibitor 5 (Sildenafil)	25 (31.2%)	11 (12.1%)	0.004
Vasoactive medication	73 (91.2%)	73 (80.2%)	0.08
Duration vasoactive medication (days) (in survivors only)	8.0 (4.3–19.0)	6.0 (3.3–11.8)	0.02
Number of treatment failures	27 (33.8%)	20 (22.0%)	0.01
Presence pulmonary hypertension	57 (71.3%) Missing: n = 3	59 (64.8%) Missing: n = 4	0.16
Severity pulmonary hypertension			0.59
None	20 (25.0%)	29 (31.9%)	
<2/3 systemic	9 (11.3%)	10 (11.0%)	
2/3 systemic–systemic	26 (32.5%)	26 (28.6%)	
> systemic	22 (27.5%)	21 (23.1%)	
Missing	3 (3.8%)	5 (4.4%)	

Results presented as n (%) or median (IQR). BPD indicates bronchopulmonary dysplasia; ECMO, extracorporeal membrane oxygenation.

cardiac anomalies, and the outcome of patients who did not receive surgical therapy.

A meta-analysis of HFOV and prevention of BPD in prematurely born infants has shown a significant, although modest reduction in BPD,[25] but a subsequent more detailed analysis did not confirm that effect.[26] A recently reported follow-up study of one of the RCTs included in that meta-analysis showed superior lung function at 11 to 14 years, despite no reduction in BPD.[27] It would thus be important to re-assess our patients at school age. It is acknowledged that the lung morphology of very prematurely born infants is very different from the dysplastic lungs of CDH infants, so we cannot extrapolate those results to our study.

In conclusion, although secondary analyses seem to suggest some benefit to CMV, based on the primary outcome of interest, we must conclude that there is no difference in effect between CMV and HFO as a primary mode of ventilation in infants with antenatally diagnosed CDH. Infants with CDH initially ventilated by CMV compared with those who received HFO required a shorter duration of ventilation and vasoactive medication and were less likely to require other medication to treat pulmonary hypertension or ECMO.

ACKNOWLEDGMENTS

We thank H. te Beest (Erasmus MC-Sophia, Rotterdam, the Netherlands) for her help in setting up this trial and I. van Rijnsoever (Radboudumc, Nijmegen, the Netherlands) for her help in collecting patient data. This research was partly funded by the SSWO Willem H. Kröger foundation.

REFERENCES

1. Lally KP. Congenital diaphragmatic hernia. *Curr Opin Pediatr.* 2002;14:486–490.
2. van den Hout L, Reiss I, Felix JF, et al. Risk factors for chronic lung disease and mortality in newborns with congenital diaphragmatic hernia. *Neonatology.* 2010;98:370–380.
3. Logan JW, Cotten CM, Goldberg RN, et al. Mechanical ventilation strategies in the management of congenital diaphragmatic hernia. *Semin Pediatr Surg.* 2007;16:115–125.
4. Reiss I, Schaible T, van den Hout L, et al. Standardized postnatal management of infants with congenital diaphragmatic hernia in Europe: the CDH EURO Consortium consensus. *Neonatology.* 2010;98:354–364.
5. van den Hout L, Schaible T, Cohen-Overbeek TE, et al. Actual outcome in infants with congenital diaphragmatic hernia: the role of a standardized postnatal treatment protocol. *Fetal Diagn Ther.* 2011;29:55–63.
6. Migliazza L, Bellan C, Alberti D, et al. Retrospective study of 111 cases of congenital diaphragmatic hernia treated with early high-frequency oscillatory ventilation and presurgical stabilization. *J Pediatr Surg.* 2007; 42:1526–1532.
7. Somaschini M, Locatelli G, Salvoni L, et al. Impact of new treatments for respiratory failure on outcome of infants with congenital diaphragmatic hernia. *Eur J Pediatr.* 1999;158:780–784.
8. Ng GY, Derry C, Marston L, et al. Reduction in ventilator-induced lung injury improves outcome in congenital diaphragmatic hernia? *Pediatr Surg Int.* 2008;24:145–150.
9. Cacciari A, Ruggeri G, Mordenti M, et al. High-frequency oscillatory ventilation versus conventional mechanical ventilation in congenital diaphragmatic hernia. *Eur J Pediatr Surg.* 2001;11:3–7.
10. Miguet D, Claris O, Lapillonne A, et al. Preoperative stabilization using high-frequency oscillatory ventilation in the management of congenital diaphragmatic hernia. *Crit Care Med.* 1994;22(Suppl 9):S77–S82.
11. Reyes C, Chang LK, Waffarn F, et al. Delayed repair of congenital diaphragmatic hernia with early high-frequency oscillatory ventilation during preoperative stabilization. *J Pediatr Surg.* 1998;33:1010–1014; discussion 1014–1016.
12. van den Hout L, Tibboel D, Vijfhuize S, et al. The VICI-trial: high frequency oscillation versus conventional mechanical ventilation in newborns with congenital diaphragmatic hernia: an international multicentre randomized controlled trial. *BMC Pediatr.* 2011;11:98.
13. Jobe AH, Bancalari E. Bronchopulmonary dysplasia. *Am J Respir Crit Care Med.* 2001;163:1723–1729.
14. Lally KP, Lasky RE, Lally PA, et al. Standardized reporting for congenital diaphragmatic hernia – an international consensus. *J Pediatr Surg.* 2013; 48:2408–2415.
15. Keller RL, Tacy TA, Hendricks-Munoz K, et al. Congenital diaphragmatic hernia: endothelin-1, pulmonary hypertension, and disease severity. *Am J Respir Crit Care Med.* 2010;182:555–561.
16. van Kaam AH, de Jaegere A, Haitsma JJ, et al. Positive pressure ventilation with the open lung concept optimizes gas exchange and reduces ventilator-induced lung injury in newborn piglets. *Pediatr Res.* 2003;53: 245–253.

© 2015 Wolters Kluwer Health, Inc. All rights reserved.

Copyright © 2015 Wolters Kluwer Health, Inc. Unauthorized reproduction of this article is prohibited.

17. Imai Y, Nakagawa S, Ito Y, et al. Comparison of lung protection strategies using conventional and high-frequency oscillatory ventilation. *J Appl Physiol*. 2001;91:1836–1844.
18. Wilson JM, Lund DP, Lillehei CW, et al. Congenital diaphragmatic hernia – a tale of two cities: the Boston experience. *J Pediatr Surg*. 1997;32:401–405.
19. Papadakos BL. *Mechanical Ventilation: Clinical Applications and Pathophysiology*. Saunders Elsevier; Philadelphia 2008.
20. Dreyfuss D, Saumon G. Role of tidal volume, FRC, and end-inspiratory volume in the development of pulmonary edema following mechanical ventilation. *Am Rev Respir Dis*. 1993;148:1194–1203.
21. Schultz C, Tautz J, Reiss I, et al. Prolonged mechanical ventilation induces pulmonary inflammation in preterm infants. *Biol Neonate*. 2003;84:64–66.
22. Tibboel J, Reiss I, de Jongste JC, et al. Sphingolipids in lung growth and repair. *Chest*. 2014;145:120–128.
23. Lally KP, Lally PA, Langham MR, et al. Surfactant does not improve survival rate in preterm infants with congenital diaphragmatic hernia. *J Pediatr Surg*. 2004;39:829–833.
24. Ali K, Grigoratos D, Cornelius V, et al. Outcome of CDH infants following fetoscopic tracheal occlusion: influence of premature delivery. *J Pediatr Surg*. 2013;48:1831–1836.
25. Van Marter LJ. Strategies for preventing bronchopulmonary dysplasia. *Curr Opin Pediatr*. 2005;17:174–180.
26. Soll RF. The clinical impact of high frequency ventilation: review of the Cochrane meta-analyses. *J Perinatol*. 2006;26(Suppl 1):S38–S42; discussion S43–45.
27. Greenough A, Peacock J, Zivanovic S, et al. United Kingdom Oscillation Study: long-term outcomes of a randomised trial of two modes of neonatal ventilation. *Health Technol Assess*. 2014;18:v–xx. 1-95.

 © 2015 Wolters Kluwer Health, Inc. All rights reserved.

Copyright © 2015 Wolters Kluwer Health, Inc. Unauthorized reproduction of this article is prohibited.
