# Supplementary material for: Sphingolipids in Congenital Diaphragmatic Hernia; Results from an International Multicenter Study
Source: PLoS One. 2016 May 9;11(5):e0155136. doi: 10.1371/journal.pone.0155136 (PMC4861280; doi:10.1371/journal.pone.0155136)
Supplement: S3 File — (PDF) [file pone.0155136.s003.pdf]

PLOS

Figure file quality report: 2016-03-04				
Original Filename	PACE Filename	Status	Error Detail(s)	PACE Adjustments
Fig1		✓	• No Error	• EPS file is converted to a TIF for submission. Please inspect the tif version for image clarity and content.
